# A conjugate gradient algorithm for large-scale unconstrained optimization problems and nonlinear equations

**DOI:** 10.1186/s13660-018-1703-1

**Published:** 2018-05-11

**Authors:** Gonglin Yuan, Wujie Hu

**Affiliations:** College of Mathematics and Information Science, Guangxi University, Nanning, P.R. China

**Keywords:** 90C26, Conjugate gradient, Descent property, Global convergence

## Abstract

For large-scale unconstrained optimization problems and nonlinear equations, we propose a new three-term conjugate gradient algorithm under the Yuan–Wei–Lu line search technique. It combines the steepest descent method with the famous conjugate gradient algorithm, which utilizes both the relevant function trait and the current point feature. It possesses the following properties: (i) the search direction has a sufficient descent feature and a trust region trait, and (ii) the proposed algorithm globally converges. Numerical results prove that the proposed algorithm is perfect compared with other similar optimization algorithms.

## Introduction

It is well known that the model of small- and medium-scale smooth functions is simple since it has many optimization algorithms, such as Newton, quasi-Newton, and bundle algorithms. Note that three algorithms fail to effectively address large-scale optimization problems because they need to store and calculate relevant matrices, whereas the conjugate gradient algorithm is successful because of its simplicity and efficiency.

The optimization model is an important mathematic problem since it has been applied to various fields such as economics, engineering, and physics (see [1–12]). Fletcher and Reeves [13] successfully address large-scale unconstrained optimization problems on the basis of the conjugate gradient algorithm and obtained amazing achievements. The conjugate gradient algorithm is increasingly famous because of its simplicity and low requirement of calculation machine. In general, a good conjugate gradient algorithm optimization algorithm includes a good conjugate gradient direction and an inexact line search technique (see [14–18]). At present, the conjugate gradient algorithm is mostly applied to smooth optimization problems, and thus, in this paper, we propose a modified LS conjugate gradient algorithm to solve large-scale nonlinear equations and smooth problems. The common algorithms of addressing nonlinear equations include Newton and quasi-Newton methods (see [19–21]), gradient-based, CG methods (see [22–24]), trust region methods (see [25–27]), and derivative-free methods (see [28]), and all of them fail to address large-scale problems. The famous optimization algorithms of spectral gradient approach, limited-memory quasi-Newton method and conjugate gradient algorithm, are suitable to solve large-scale problems. Li and Li [29] proposed various algorithms on the basis of modified PRP conjugate gradient, which successfully solve large-scale nonlinear equations.

A famous mathematic model is given by

1.1 $$ \min \bigl\{ f(x) \mid x \in \Re^{n} \bigr\} , $$

where $f: \Re^{n}\rightarrow \Re $ and $f\in C^{2}$. The relevant model is widely used in life and production. However, it is a complex mathematic model since it needs to meet various conditions in the field [30–33]. Experts and scholars have conducted numerous in-depth studies and have made some significant achievements (see [14, 34, 35]). It is well known that the steepest descent algorithm is perfect since it is simple and its computational and memory requirements are low. It is regrettable that the steepest descent method sometimes fails to solve problems due to the “sawtooth phenomenon”. To overcome this flaw, experts and scholars presented an efficient conjugate gradient method, which provides high performance with a simple form. In general, the mathematical formula for (1.1) is

1.2 $$ x_{k+1}=x_{k}+\alpha_{k}d_{k},\quad k \in \{0, 1, 2,\dots \}, $$

where $x_{k+1}$ is the next iteration point, $\alpha_{k}$ is the step length, and $d_{k}$ is the search direction. The famous weak Wolfe–Powell (WWP) line search technique is determined by

1.3 $$ g(x_{k}+\alpha_{k}d_{k})^{T}d_{k} \ge \rho g_{k}^{T}d_{k} $$

and

1.4 $$ f(x_{k}+\alpha_{k}d_{k}) \le f_{k}+\varphi \alpha_{k}g_{k}^{T}d_{k}, $$

where $\varphi \in (0, 1/2)$, $\alpha_{k} > 0$, and $\rho \in ( \varphi, 1)$. The direction $d_{k+1}$ is often defined by the formula

1.5 $$\begin{aligned} d_{k+1}=\textstyle\begin{cases} -g_{k+1}+\beta_{k}d_{k} & \mbox{if } k\geq 1, \\ -g_{k+1}& \mbox{if } k=0, \end{cases}\displaystyle \end{aligned}$$

where $\beta_{k} \in \Re $. An increasing number of efficient conjugate gradient algorithms have been proposed by different expressions of $\beta_{k}$ and $d_{k}$ (see [13, 36–42] etc.). The well-known PRP algorithm is given by

1.6 $$ \beta_{k}^{\mathrm{PRP}}=\frac{g_{k+1}^{T}(g_{k+1}-g_{k})}{\Vert g_{k}\Vert \Vert g_{k}\Vert }, $$

where $g_{k}$, $g_{k+1}$, and $f_{k}$ denote $g(x_{k})$, $g(x_{k+1})$, and $f(x_{k})$, respectively; $g_{k+1}=g(x_{k+1})=\nabla f(x_{k+1})$ is the gradient function at the point $x_{k+1}$. It is well known that the PRP algorithm is efficient but has shortcomings, as it does not possess global convergence under the WWP line search technique. To solve this complex problem, Yuan, Wei, and Lu [43] developed the following creative formula (YWL) for the normal WWP line search technique and obtained many fruitful theories:

1.7 $$ f(x_{k}+\alpha_{k}d_{k}) \leq f(x_{k})+\iota \alpha_{k}g_{k}^{T}d_{k}+ \alpha_{k}\min \bigl[-\iota_{1}g_{k}^{T}d_{k}, \iota \alpha_{k}\Vert d_{k}\Vert ^{2}/2\bigr] $$

and

1.8 $$ g(x_{k}+\alpha_{k}d_{k})^{T}d_{k} \geq \tau g_{k}^{T}d_{k}+\min \bigl[- \iota_{1}g_{k}^{T}d_{k},\iota \alpha_{k}\Vert d_{k}\Vert ^{2}\bigr], $$

where $\iota \in (0,\frac{1}{2})$, $\alpha_{k} > 0$, $\iota_{1} \in (0,\iota)$, and $\tau \in (\iota,1)$. Further work can be found in [24]. Based on the innovation of YWL line search technique, Yuan pay much attention to normal Armijo line search technique and make further study. They proposed an efficient modified Armijo line search technique:

1.9 $$ f(x_{k}+\alpha_{k}d_{k}) \le f(x_{k})+\lambda \alpha_{k}g_{k}^{T}d _{k}+\alpha_{k}\min \biggl[-\lambda_{1}g_{k}^{T}d_{k}, \lambda \frac{\alpha _{k}}{2}\Vert d_{k}\Vert ^{2}\biggr], $$

where $\lambda, \gamma \in (0,1)$, $\lambda_{1} \in (0,\lambda)$, and $\alpha_{k}$ is the largest number of $\{\gamma^{k}|k=0,1,2,\ldots \}$. In addition, experts and scholars pay much attention to the three-term conjugate gradient formula. Zhang et al. [44] proposed the famous formula

1.10 $$ d_{k+1}=-g_{k+1} + \frac{g_{k+1}^{T}y_{k}d_{k}-d_{k}^{T}g_{k+1}y_{k}}{g _{k}^{T}g_{k}}. $$

Nazareth [45] proposed the new formula

1.11 $$ d_{k+1}=-y_{k}+\frac{y_{k}^{T}y_{k}}{y_{k}^{T}d_{k}}d_{k}+ \frac{y_{k-1} ^{T}y_{k}}{y_{k-1}^{T}d_{k-1}}d_{k-1}, $$

where $y_{k}=g_{k+1}-g_{k}$ and $s_{k}=x_{k+1}-x_{k}$. These two conjugate gradient methods have a sufficient descent property but fail to have the trust region feature. To improve these methods, Yuan et al. [46, 47] make a further study and get some good results. This inspires us to continue the study and extend the conjugate gradient methods to get better results. In this paper, motivated by in-depth discussions, we express a modified conjugate gradient algorithm, which has the following properties:

- The search direction has a sufficient descent feature and a trust region trait.
- Under mild assumptions, the proposed algorithm possesses the global convergence.
- The new algorithm combines the steepest descent method with the conjugate gradient algorithm.
- Numerical results prove that it is perfect compared to other similar algorithms.

The rest of the paper is organized as follows. The next section presents the necessary properties of the proposed algorithm. The global convergence is stated in Sect. 3. In Sect. 4, we report the corresponding numerical results. In Sect. 5, we introduce the large-scale nonlinear equations and express the new algorithm. Some necessary properties are listed in Sect. 6. The numerical results are reported in Sect. 7. Without loss of generality, $f(x_{k})$ and $f(x_{k+1})$ are replaced by $f_{k}$ and $f_{k+1}$, and $\|\cdot \|$ is the Euclidean norm.

## New modified conjugate gradient algorithm

Experts and scholars have conducted thorough research on the conjugate gradient algorithm and have obtained rich theoretical achievements. In light of the previous work by experts on the conjugate gradient algorithm, a sufficient descent feature is necessary for the global convergence. Thus, we express a new conjugate gradient algorithm under the YWL line search technique as follows:

2.1 $$\begin{aligned} d_{k+1}=\textstyle\begin{cases} -\eta_{1}g_{k+1}+(1-\eta_{1})(d_{k}^{T}g_{k+1}y_{k}^{*}-g_{k+1}^{T}y _{k}^{*}d_{k})/\delta & \mbox{if } k \ge 1, \\ -g_{k+1} & \mbox{if } k = 0, \end{cases}\displaystyle \end{aligned}$$

where $\delta =\max (\min (\eta_{5}|s_{k}^{T}y_{k}^{*}|,|d_{k}^{T}y _{k}^{*}|),\eta_{2}\|y_{k}^{*}\|\|d_{k}\|,\eta_{3}\|g_{k}\|^{2})+\eta _{4}*\|d_{k}\|^{2}$, $y_{k}^{*}=g_{k+1}-\frac{\|g_{k+1}\|^{2}}{\|g _{k}\|^{2}}g_{k}$, and $\eta_{i} >0$ ($i=1, 2,3, 4, 5$). The search direction is well defined, and its properties are stated in the next section. Now, we introduce a new conjugate gradient algorithm called Algorithm 2.1.

**Algorithm 2.1:**
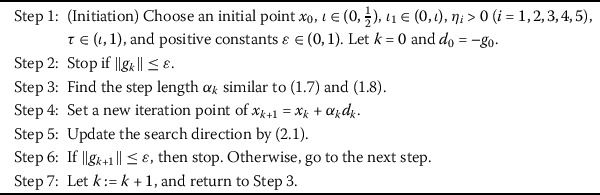
Modified three-term conjugate gradient algorithm for optimization model

## Important characteristics

This section lists some important properties of sufficient descent, the trust region, and the global convergence of Algorithm 2.1. It expresses the necessary proof.

### Lemma 3.1

*If search direction* $d_{k}$ *meets condition of* (2.1), *then*

3.1 $$ g_{k}^{T}d_{k}=-\eta_{1} \Vert g_{k}\Vert ^{2} $$

*and*

3.2 $$ \Vert d_{k}\Vert \leq \bigl(\eta_{1}+2(1- \eta_{1})/\eta_{2}\bigr)\Vert g_{k}\Vert . $$

### Proof

It is obvious that formulas of (3.1) and (3.2) are true for $k=0$.

Now consider the condition $k \geq 1$. Similarly to (2.1), we have

$$\begin{aligned} g_{k+1}^{T}d_{k+1} =&g_{k+1}^{T}\bigl[-\eta_{1}g_{k+1}+(1- \eta_{1}) \bigl(d_{k} ^{T}g_{k+1}y_{k}^{*}-g_{k+1}^{T}y_{k}^{*}d_{k} \bigr)/\delta \bigr] \\ =& -\eta_{1}\Vert g_{k+1}\Vert ^{2}+(1- \eta_{1}) \bigl(g_{k+1}^{T}d_{k}^{T}g_{k+1}y _{k}^{*}-g_{k+1}^{T}g_{k+1}^{T}y_{k}^{*}d_{k} \bigr)/\delta \\ =& -\eta_{1}\Vert g_{k+1}\Vert ^{2} \end{aligned}$$

and

$$\begin{aligned} \Vert d_{k+1}\Vert =&\bigl\Vert - \eta_{1}g_{k+1}+(1-\eta_{1}) \bigl(d_{k}^{T}g_{k+1}y_{k}^{*}-g_{k+1}^{T}y_{k}^{*}d_{k} \bigr)/\delta \bigr\Vert \\ \leq & \eta_{1}\Vert g_{k+1}\Vert +2(1- \eta_{1})\Vert g_{k+1}\Vert \bigl\Vert y_{k}^{*}\bigr\Vert \Vert d_{k}\Vert /\delta \\ \leq & \eta_{1}\Vert g_{k+1}\Vert +2(1- \eta_{1})\Vert g_{k+1}\Vert \bigl\Vert y_{k}^{*}\bigr\Vert \Vert d_{k}\Vert /\bigl( \eta_{2} \bigl\Vert y_{k}^{*}\bigr\Vert \Vert d_{k}\Vert \bigr) \\ =&\bigl(\eta_{1}+2(1-\eta_{1})/\eta_{2}\bigr) \Vert g_{k+1}\Vert . \end{aligned}$$

Thus, the statement is proved. □

Similarly to (3.1) and (3.2), the algorithm has a sufficient descent feature and a trust region trait. To obtain the global convergence, we propose the following necessary assumptions.

### Assumption 1

(i) The level set of $\pi =\{x|f(x) \leq f(x _{0})\}$ is bounded.
(ii) The objective function $f \in C^{2}$ is bounded from below, and its gradient function *g* is Lipschitz continuous, thats is, there exists a constant *ζ* such that
  3.3 $$ \bigl\Vert g(x)-g(y)\bigr\Vert \leq \zeta \Vert x-y\Vert ,\quad x, y \in R^{n}. $$
  The existence and necessity of the step length $\alpha_{k}$ are established in [43]. In view of the discussion and established technique, the global convergence of the proposed algorithm is expressed as follows.

### Theorem 3.1

*If Assumptions* (i)–(ii) *are satisfied and the relative sequences of* $\{x_{k}\}$, $\{d_{k}\}$, $\{g_{k}\}$, *and* $\{\alpha_{k}\}$ *are generated by Algorithm* 2.1, *then*

3.4 $$ \lim_{k \rightarrow \infty } \Vert g_{k}\Vert =0. $$

### Proof

By (1.7), (3.1), and (3.3) we have

$$\begin{aligned} f(x_{k}+\alpha_{k}d_{k}) \leq & f_{k}+ \iota \alpha_{k}g_{k}^{T}d_{k}+ \alpha_{k}\min \bigl[-\iota_{1}g_{k}^{T}d_{k}, \iota \alpha_{k}\Vert d_{k}\Vert ^{2}/2\bigr] \\ \leq & f_{k}+\iota \alpha_{k}g_{k}^{T}d_{k}- \alpha_{k}\iota_{1}g_{k} ^{T}d_{k} \\ \leq & f_{k}+\alpha_{k}(\iota -\iota_{1})g_{k}^{T}d_{k} \\ \leq & f_{k}-\eta_{1}\alpha_{k}(\iota - \iota_{1})\Vert g_{k}\Vert ^{2}. \end{aligned}$$

Summing these inequalities from $k=0$ to ∞, under Assumption (ii), we obtain

3.5 $$ \sum_{k=0}^{\infty } \eta_{1}\alpha_{k}(\iota -\iota_{1})\Vert g_{k}\Vert ^{2} \leq f(x_{0})-f_{\infty }< + \infty. $$

This means that

3.6 $$ \lim_{k \rightarrow \infty }\alpha_{k}\Vert g_{k}\Vert ^{2}=0. $$

Similarly to (1.8) and (3.1), we obtain

$$\begin{aligned} g(x_{k}+\alpha_{k}d_{k})^{T}d_{k} \geq & \tau g_{k}^{T}d_{k}+\min \bigl[- \iota_{1}g_{k}^{T}d_{k},\iota \alpha_{k}\Vert d_{k}\Vert ^{2}\bigr] \\ \geq & \tau g_{k}^{T}d_{k}. \end{aligned}$$

Thus, we obtain the following inequality:

$$\begin{aligned} -\eta_{1}(\tau -1)\Vert g_{k}\Vert ^{2} \leq & (\tau -1)g_{k}^{T}d_{k} \\ \leq & \bigl[g(x_{k}+\alpha_{k}d_{k})-g(x_{k}) \bigr]^{T}d_{k} \\ \leq & \bigl\Vert g(x_{k}+\alpha_{k}d_{k})-g(x_{k}) \bigr\Vert \Vert d_{k}\Vert \\ \leq & \alpha_{k}\zeta \Vert d_{k}\Vert ^{2}, \end{aligned}$$

where the last inequality is obtained since the gradient function is Lipschitz continuous. Then, we have

$$\alpha_{k} \geq \frac{(1-\tau)\eta_{1}\Vert g_{k}\Vert ^{2}}{\zeta \Vert d_{k}\Vert ^{2}} \geq \frac{(1-\tau)\eta_{1}\Vert g_{k}\Vert ^{2}}{(\zeta (\eta_{1}+2(1- \eta_{1})/\eta_{2})^{2}\Vert g_{k}\Vert ^{2}))}= \frac{(1-\tau)\eta_{1}}{( \zeta (\eta_{1}+2(1-\eta_{1})/\eta_{2})^{2})}. $$

By (3.6) we arrive at the conclusion

$$\lim_{k \rightarrow \infty } \Vert g_{k}\Vert ^{2}=0, $$

as claimed. □

## Numerical results

In this section, we list the numerical result in terms of the algorithm characteristics NI, NFG, and CPU, where NI is the total iteration number, NFG is the sum of the calculation frequency of the objective function and gradient function, and CPU is the calculation time in seconds.

### Problems and test experiments

The tested problems listed in Table 1 stem from [48]. At the same time, we introduce two different algorithms into this section to measure the objective algorithm efficiency through the tested problems. We denote the two algorithms as Algorithm 2 and Algorithm 3. They are different from Algorithm 2.1 only at Step 5. One is determined by (1.10), and the other is computed by (1.11).

**Table 1:** Test problems

No.	Problem
1	Extended Freudenstein and Roth Function
2	Extended Trigonometric Function
3	Extended Rosenbrock Function
4	Extended White and Holst Function
5	Extended Beale Function
6	Extended Penalty Function
7	Perturbed Quadratic Function
8	Raydan 1 Function
9	Raydan 2 Function
10	Diagonal 1 Function
11	Diagonal 2 Function
12	Diagonal 3 Function
13	Hager Function
14	Generalized Tridiagonal 1 Function
15	Extended Tridiagonal 1 Function
16	Extended Three Exponential Terms Function
17	Generalized Tridiagonal 2 Function
18	Diagonal 4 Function
19	Diagonal 5 Function
20	Extended Himmelblau Function
21	Generalized PSC1 Function
22	Extended PSC1 Function
23	Extended Powell Function
24	Extended Block Diagonal BD1 Function
25	Extended Maratos Function
26	Extended Cliff Function
27	Quadratic Diagonal Perturbed Function
28	Extended Wood Function
29	Extended Hiebert Function
30	Quadratic Function QF1 Function
31	Extended Quadratic Penalty QP1 Function
32	Extended Quadratic Penalty QP2 Function
33	A Quadratic Function QF2 Function
34	Extended EP1 Function
35	Extended Tridiagonal-2 Function
36	BDQRTIC Function (CUTE)
37	TRIDIA Function (CUTE)
38	ARWHEAD Function (CUTE)
38	ARWHEAD Function (CUTE)
40	NONDQUAR Function (CUTE)
41	DQDRTIC Function (CUTE)
42	EG2 Function (CUTE)
43	DIXMAANA Function (CUTE)
44	DIXMAANB Function (CUTE)
45	DIXMAANC Function (CUTE)
46	DIXMAANE Function (CUTE)
47	Partial Perturbed Quadratic Function
48	Broyden Tridiagonal Function
49	Almost Perturbed Quadratic Function
50	Tridiagonal Perturbed Quadratic Function
51	EDENSCH Function (CUTE)
52	VARDIM Function (CUTE)
53	STAIRCASE S1 Function
54	LIARWHD Function (CUTE)
55	DIAGONAL 6 Function
56	DIXON3DQ Function (CUTE)
57	DIXMAANF Function (CUTE)
58	DIXMAANG Function (CUTE)
59	DIXMAANH Function (CUTE)
60	DIXMAANI Function (CUTE)
61	DIXMAANJ Function (CUTE)
62	DIXMAANK Function (CUTE)
63	DIXMAANL Function (CUTE)
64	DIXMAAND Function (CUTE)
65	ENGVAL1 Function (CUTE)
66	FLETCHCR Function (CUTE)
67	COSINE Function (CUTE)
68	Extended DENSCHNB Function (CUTE)
69	DENSCHNF Function (CUTE)
70	SINQUAD Function (CUTE)
71	BIGGSB1 Function (CUTE)
72	Partial Perturbed Quadratic PPQ2 Function
73	Scaled Quadratic SQ1 Function

*Stopping rule:* If the inequality $| f(x_{k})| > e_{1}$ is correct, let $stop1=\frac{|f(x_{k})-f(x_{k+1})|}{| f(x_{k})|}$ or $stop1=| f(x _{k})-f(x_{k+1})|$. The algorithm stops when one of the following conditions is satisfied: $\|g(x)\|<\epsilon $, the iteration number is greater than 2000, or $stop 1 < e_{2}$, where $e_{1}=e_{2}=10^{-5}$ and $\epsilon =10^{-6}$. In Table 1, “No” and “problem” represent the index of the the tested problems and the name of the problem, respectively.

*Initiation:* $\iota =0.3$, $\iota_{1}=0.1$, $\tau =0.65$, $\eta_{1}=0.65$, $\eta_{2}=0.001$, $\eta_{3}=0.001$, $\eta_{4}=0.001$, $\eta_{5}=0.1$.

*Dimension:* 1200, 3000, 6000, 9000.

*Calculation environment:* The calculation environment is a computer with 2 GB of memory, a Pentium(R) Dual-Core CPU E5800@3.20 GHz, and the 64-bit Windows 7 operation system.

A list of the numerical results with the corresponding problem index is listed in Table 2. Then, based on the technique in [49], the plots of the corresponding figures are presented for the three discussed algorithms.

**Table 2:** Numerical results

NO	Dim	Algorithm 2.1			Algorithm 2			Algorithm 3		
		NI	NFG	CPU	NI	NFG	CPU	NI	NFG	CPU
1	9000	4	20	0.124801	14	48	0.405603	5	26	0.249602
2	9000	71	327	1.965613	27	89	0.670804	32	136	0.858005
3	9000	7	20	0.0312	37	160	0.249602	27	147	0.202801
4	9000	12	49	0.280802	34	161	0.717605	42	219	0.951606
5	9000	13	56	0.202801	20	63	0.249602	5	24	0.0624
6	9000	65	252	0.421203	43	143	0.280802	3	9	0.0312
7	9000	11	37	0.0624	478	979	2.215214	465	1479	2.558416
8	9000	5	20	0.0624	22	55	0.156001	14	54	0.156001
9	9000	6	16	0.0312	5	21	0.0624	3	8	0.0312
10	9000	2	13	0.0156	2	13	0.000001	2	13	0.000001
11	9000	3	17	0.0312	7	34	0.0624	17	87	0.218401
12	9000	3	10	0.0312	19	40	0.202801	14	50	0.202801
13	9000	3	24	0.0624	3	24	0.0312	3	24	0.0156
14	9000	4	12	4.305628	5	14	5.382034	5	14	5.226033
15	9000	19	77	9.984064	22	66	9.516061	21	71	10.296066
16	9000	3	11	0.0624	6	27	0.078	6	18	0.0624
17	9000	11	45	0.374402	27	69	0.780005	27	87	0.811205
18	9000	5	23	0.0312	3	10	0.000001	3	10	0.0312
19	9000	3	9	0.0624	3	9	0.0312	3	19	0.0312
20	9000	19	76	0.124801	15	36	0.0624	3	9	0.0312
21	9000	12	47	0.156001	13	61	0.187201	15	59	0.218401
22	9000	7	46	0.795605	8	70	0.577204	6	46	0.686404
23	9000	9	45	0.218401	101	357	2.090413	46	150	0.873606
24	9000	5	47	0.093601	14	88	0.156001	14	97	0.249602
25	9000	9	28	0.0312	40	214	0.249602	8	46	0.0624
26	9000	24	102	0.327602	24	100	0.249602	3	24	0.0312
27	9000	6	20	0.0312	34	109	0.187201	92	321	0.530403
28	9000	13	50	0.124801	20	83	0.109201	23	84	0.140401
29	9000	6	36	0.0468	4	21	0.0312	4	21	0.0312
30	9000	11	37	0.0624	454	931	1.450809	424	1346	1.747211
31	9000	18	63	0.124801	15	51	0.093601	3	10	0.0312
32	9000	18	70	0.218401	23	61	0.218401	3	18	0.0624
33	9000	2	5	0.000001	2	5	0.0312	2	5	0.000001
34	9000	8	16	0.0312	6	12	0.0312	3	6	0.0312
35	9000	4	13	0.0312	4	10	0.0312	3	8	0.000001
36	9000	7	23	4.602029	8	28	5.569236	10	47	8.673656
37	9000	7	23	0.0624	1412	2829	6.942044	2000	6021	11.356873
38	9000	4	18	0.0312	8	35	0.187201	4	11	0.0312
39	9000	5	19	0.0312	28	56	0.124801	3	8	0.0312
40	9000	13	43	0.561604	835	2936	36.223432	9	41	0.421203
41	9000	10	32	0.0624	17	41	0.093601	22	81	0.124801
42	9000	4	33	0.0624	13	35	0.124801	9	47	0.109201
43	9000	16	62	1.029607	16	38	0.951606	13	48	0.780005
44	9000	3	17	0.156001	9	50	0.624004	3	17	0.187201
45	9000	21	118	1.49761	12	81	0.858006	3	24	0.202801
46	9000	20	81	1.435209	209	443	11.247672	110	362	6.630042
47	9000	11	37	27.066173	30	97	68.64044	37	112	87.220159
48	9000	13	54	9.718862	31	92	18.610919	23	50	11.980877
49	9000	11	37	0.0624	478	979	1.51321	504	1592	1.887612
50	9000	11	37	7.971651	472	967	263.68849	444	1273	299.381519
51	9000	6	31	0.156001	7	25	0.218401	3	17	0.124801
52	9000	62	186	0.998406	63	195	0.842405	4	21	0.0624
53	9000	10	32	0.0312	2000	4059	7.72205	1865	5618	7.971651
54	9000	4	11	0.0312	21	79	0.156001	17	79	0.124801
55	9000	10	24	3.010819	7	25	3.213621	3	10	1.076407
56	9000	7	21	0.0156	2000	4003	6.489642	1390	4107	5.335234
57	9000	5	39	0.358802	67	220	4.024826	3	24	0.202801
58	9000	5	24	0.343202	114	282	6.411641	82	315	5.257234
59	9000	5	39	0.343202	68	310	4.72683	3	23	0.171601
60	9000	18	74	1.294808	206	437	11.107271	119	363	6.957645
61	9000	5	39	0.358802	85	247	4.929632	3	24	0.218401
62	9000	4	32	0.234001	4	32	0.249602	3	22	0.187201
63	9000	3	22	0.187201	3	22	0.187201	3	22	0.187201
64	9000	5	39	0.343202	23	147	1.747211	3	23	0.218401
65	9000	12	59	15.334898	14	51	14.944896	7	21	6.130839
66	9000	3	9	1.62241	2000	4022	1114.767546	529	2196	443.526443
67	9000	5	28	0.093601	15	58	0.280802	3	23	0.0312
68	9000	13	55	0.109201	11	27	0.0624	9	25	0.0624
69	9000	16	73	0.218401	24	55	0.187201	20	70	0.171601
70	9000	4	13	2.542816	41	203	36.332633	35	231	37.783442
71	9000	11	35	0.093601	2000	4014	6.708043	1491	4631	5.600436
72	9000	9	30	21.85574	1089	3897	2675.588751	287	1015	704.391315
73	9000	19	65	0.093601	607	1269	1.856412	669	2062	2.293215

**Table 3:** Test problems

No.	Problem
1	Exponential function 1
2	Exponential function 2
3	Trigonometric function
4	Singular function
5	Logarithmic function
6	Broyden tridiagonal function
7	Trigexp function
8	Strictly convex function 1
9	Strictly convex function 2
10	Zero Jacobian function
11	Linear function-full rank
12	Penalty function
13	Variable dimensioned function
14	Extended Powel singular function
15	Tridiagonal system
16	Five-diagonal system
17	Extended Freudentein and Roth function
18	Extended Wood problem
19	Discrete boundary value problem

*Other case:* To save the paper space, we only list the data of dimension of 9000, and the remaining data are listed in the attachment.

### Results and discussion

**Figure 1:**
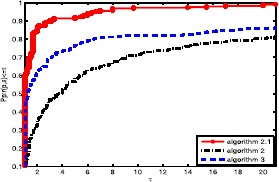
Performance profiles of these methods (NI)

**Figure 2:**
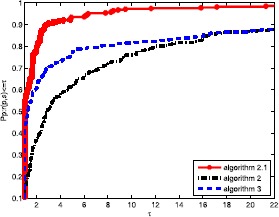
Performance profiles of these methods (NFG)

**Figure 3:**
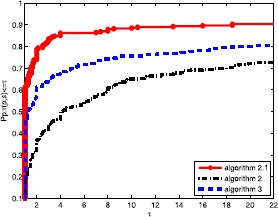
Performance profiles of these methods (CPU time)

Obviously, the objective algorithm (Algorithm 2.1) is more effective than the other algorithms since the point value on the algorithm curve is largest among the three curves. In Fig. 1, the proposed algorithm curve is above the other curves. This means that the objective algorithm solves complex problems with fewer iterations, and Algorithm 3 is better than Algorithm 2. In Fig. 2, we obtain that the proposed algorithm has a large initial point, which means that it has high efficiency and its curve seems smoother than others. It is well known that the most important metric of an algorithm is the calculation time (CPU time), which is an essential aspect to measure the efficiency of an algorithm. Based on Fig. 3, the objective algorithm successfully fully utilizes its outstanding characteristics. Therefore, it saves time compared to the other algorithms in addressing complex problems.

## Nonlinear equations

The model of nonlinear equations is given by

5.1 $$ h(x)=0, $$

where the function of *h* is continuously differentiable and monotonous, and $x \in R^{n}$, that is,

$$\bigl(h(x)-h(y)\bigr) (x-y)>0, \quad \forall x, y \in R^{n}. $$

Scholars and writers paid much attention to this model since it significantly influences various fields such as physics and computer technology (see [1–3, 8–11]), and it has resulted in many fruitful theories and good techniques (see [47, 50–54]). By mathematical calculations we obtain that (5.1) is equivalent to the model

5.2 $$ \min F(x), $$

where $F(x)=\frac{\|h(x)\|^{2}}{2}$, and $\|\cdot \|$ is the Euclidean norm. Then, we pay much attention to the mathematical model (5.2) since (5.1) and (5.2) have the same solution. In general, the mathematical formula for (5.2) is $x_{k+1}=x_{k}+\alpha_{k}d_{k}$. Now, we introduce the following famous line search technique into this paper [47, 55]:

5.3 $$ -h(x_{k}+\alpha_{k}d_{k})^{T}d_{k} \geq \sigma \alpha_{k}\bigl\Vert h(x_{k}+ \alpha_{k}d_{k})\bigr\Vert \Vert d_{k}\Vert ^{2}, $$

where $\alpha_{k}=\max \{s, s\rho, s\rho^{2}, \ldots\}$, $s, \rho >0$, $\rho \in (0,1)$, and $\sigma >0$. Solodov [56] proposes a projection proximal point algorithm in a Hilbert space that finds the zeros of set-valued maximal monotone operators. Ceng and Yao [57–60] paid much attention to the research in Hilbert spaces and obtained successful achievements. Solodov and Svaiter [61] applied the projection technique to large-scale nonlinear equations and obtained some ideal achievements. For the projection-based technique, the famous formula

$$h(w_{k})^{T}(x_{k}-w_{k}) > 0 $$

is flexible, where $w_{k}=x_{k}+\alpha_{k}d_{k}$. The search direction is extremely important for the proposed algorithm since it largely determines the efficiency. Likewise, the algorithm contains the perfect line search technique. By the monotonicity of $h(x)$ we obtain

$$h(w_{k})^{T}\bigl(x^{*}-w_{k}\bigr) \leq 0, $$

where $x^{*}$ is the solution of $h(x^{*})=0$. We consider the hyperplane

5.4 $$ \Lambda =\bigl\{ x \in R^{n}\vert h(w_{k})^{T}(x-w_{k})=0 \bigr\} . $$

It is obvious that the hyperplane separates the current iteration point of $x_{k}$ from the zeros of the mathematical model (5.1). Then, we need to calculate the next iteration point $x_{k+1}$ through projection of current point $x_{k}$. Therefore, we give the following formula for the next point:

5.5 $$ x_{k+1}=x_{k}-\frac{h(w_{k})^{T}(x_{k}-w_{k})h(w_{k})}{\Vert h(w_{k})^{2}\Vert }. $$

In [55], it is proved that formula (5.5) is effective since it not only obtains perfect numerical results but also has perfect theoretical characteristics. Thus, we introduce it here. The formula of the search direction $d_{k+1}$ is given by

5.6 $$\begin{aligned} d_{k+1}=\textstyle\begin{cases} -\eta_{1}h_{k+1}+(1-\eta_{1})(d_{k}^{T}h_{k+1}y_{k}^{*}-h_{k+1}^{T}y _{k}^{*}d_{k})/\delta & \mbox{if } k \ge 1, \\ -h_{k+1} & \mbox{if } k = 0, \end{cases}\displaystyle \end{aligned}$$

where $\delta =\max (\min (\eta_{5}|s_{k}^{T}y_{k}^{*}|,|d_{k}^{T}y _{k}^{*}|),\eta_{2}\|y_{k}^{*}\|\|d_{k}\|,\eta_{3}\|g_{k}\|^{2})+\eta _{4}*\|d_{k}\|^{2}$, $y_{k}^{*}=h_{k+1}-\frac{\|h_{k+1}\|^{2}}{\|h _{k}\|^{2}}h_{k}$, and $\eta_{i} >0$ ($i=1, 2,3$). Now, we express the specific content of the proposed algorithm.

## The global convergence of Algorithm 5.1

First, we make the following necessary assumptions.

### Assumption 2

(i) The objective model of (5.1) has a nonempty solution set.
(ii) The function *h* is Lipschitz continuous on $R^{n}$, which means that there is a positive constant *L* such that
  6.1 $$ \bigl\Vert h(x)-h(y)\bigr\Vert \leq L\Vert x-y\Vert , \quad \forall x, y \in R^{n}. $$

By Assumption 2(ii) it is obvious that

6.2 $$ \Vert h_{k}\Vert \leq \theta, $$

where *θ* is a positive constant. Then, the necessary properties of the search direction are the following (we omit the proof):

6.3 $$ h_{k}^{T}d_{k}=-\eta_{1} \Vert h_{k}\Vert \Vert h_{k}\Vert $$

and

6.4 $$ \Vert d_{k}\Vert \leq \bigl(\eta_{1}+2(1- \eta_{1})/\eta_{2}\bigr)\Vert h_{k}\Vert . $$

Now, we give some lemmas, which we utilize to obtain the global convergence of the proposed algorithm.

### Lemma 6.1

*If Assumption* 2 *holds*, *the relevant sequence* $\{x_{k}\}$ *is produced by Algorithm *5.1, *and the point* $x^{*}$ *is the solution of the objective model* (5.1). *We obtain that the formula*

$$\bigl\Vert x_{k+1}-x^{*}\bigr\Vert ^{2} \leq \bigl\Vert x_{k}-x^{*}\bigr\Vert ^{2}-\Vert x_{k+1}-x_{k}\Vert ^{2} $$

*is correct and the sequence* $\{x_{k}\}$ *is bounded*. *Furthermore*, *either the last iteration point is the solution of the objective model and the sequence of* $\{x_{k}\}$ *is bounded*, *or the sequence of* $\{x_{k}\}$ *is infinite and satisfies the condition*

$$\sum_{k=0}^{\infty }\Vert x_{k+1}-x_{k} \Vert ^{2} < \infty. $$

**Algorithm 5.1:**
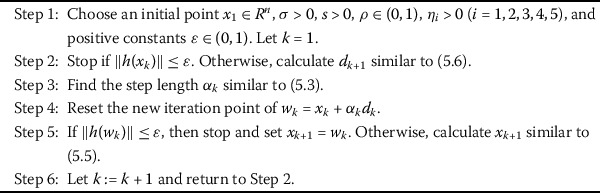
Modified three-term conjugate gradient algorithm for large-scale nonlinear equations

This paper merely proposes, but omits, the relevant proof since it is similar to the proof in [61].

### Lemma 6.2

*Algorithm *5.1 *generates an iteration point in a finite number of iteration steps*, *which satisfies the formula of* $x_{k+1}=x_{k}+\alpha _{k}d_{k}$ *if Assumption* 2 *holds*.

### Proof

We denote $\Psi = N \cup \{0\}$. We suppose that Algorithm 5.1 has terminated or the formula $\|h_{k}\| \rightarrow 0$ is erroneous. This means that there exists a constant $\varepsilon _{*}$ such that

6.5 $$ \Vert h_{k}\Vert \geq \varepsilon_{*},\quad k \in \Psi. $$

We prove this conclusion by contradiction. Suppose that certain iteration indexes $k^{*}$ fail to meet the condition (5.3) of the line search technique. Without loss of generality, we denote the corresponding step length as $\alpha_{k^{*}}^{(l)}$, where $\alpha _{k^{*}}^{(l)}=\rho^{l}s$. This means that

$$-h\bigl(x_{k^{*}}+\alpha_{k^{*}}^{(l)}d_{k^{*}} \bigr)^{T}d_{k^{*}} < \sigma \alpha_{k^{*}}^{(l)} \bigl\Vert h\bigl(x_{k^{*}}+\alpha_{k^{*}}^{(l)}d_{k^{*}} \bigr)\bigr\Vert \Vert d_{k^{*}}\Vert ^{2}. $$

By (6.3) and Assumption 2(ii) we obtain

$$\begin{aligned} \Vert h_{k^{*}}\Vert ^{2} =& - \eta_{1}h_{k^{*}}^{T}d_{k^{*}} \\ =& \eta_{1}\bigl[\bigl(h\bigl(x_{k^{*}}+\alpha_{k^{*}}^{(l)}d_{k^{*}} \bigr)-h(x_{k^{*}})\bigr)^{T}d _{k^{*}}-\bigl(h \bigl(x_{k^{*}}+\alpha_{k^{*}}^{(l)}d_{k^{*}} \bigr)^{T}d_{k^{*}}\bigr)\bigr] \\ < & \eta_{1}\bigl[L+\sigma \bigl\Vert h\bigl(x_{k^{*}}+ \alpha_{k^{*}}^{(l)}d_{k^{*}}\bigr)\bigr\Vert \bigr] \alpha_{k^{*}}^{(l)}\Vert d_{k^{*}}\Vert ^{2}, \quad \forall l \in \Psi. \end{aligned}$$

By (6.3) and (6.4) we have

$$\begin{aligned} \bigl\Vert h\bigl(x_{k^{*}}+\alpha_{k^{*}}^{(l)}d_{k^{*}} \bigr)\bigr\Vert \leq & \bigl\Vert h\bigl(x_{k^{*}}+ \alpha_{k^{*}}^{(l)}d_{k^{*}}\bigr)-h(x_{k^{*}}) \bigr\Vert +\bigl\Vert h(x_{k^{*}})\bigr\Vert \\ \leq & L\alpha_{k^{*}}^{(l)}\Vert d_{k^{*}}\Vert + \theta \\ \leq & Ls\theta \bigl(\eta_{1}+2(1-\eta_{1})/ \eta_{2}\bigr)+\theta. \end{aligned}$$

By (6.6) we obtain

$$\begin{aligned} \alpha_{k^{*}}^{(l)} >& \frac{\Vert h_{k^{*}}\Vert ^{2}}{\eta_{1}[L+\sigma \Vert h(x_{k^{*}}+\alpha_{k^{*}}^{(l)}d_{k^{*}})\Vert ]\Vert d_{k^{*}}\Vert ^{2} } \\ >&\frac{\Vert h_{k^{*}}\Vert ^{2}}{\eta_{1}[L+\sigma (Ls\theta (\eta_{1}+2(1- \eta_{1})/\eta_{2})+\theta)]\Vert d_{k^{*}}\Vert ^{2} } \\ >& \frac{\eta_{2}^{2}}{\eta_{1}[L+\sigma (Ls\theta (\eta_{1}+2/\eta _{3})+\theta)](2(1-\eta_{1})+\eta_{2}\eta_{1})^{2}},\quad \forall l \in \Psi. \end{aligned}$$

It is obvious that this formula fails to meet the definition of the step length $\alpha_{k^{*}}^{(l)}$. Thus, we conclude that the proposed line search technique is reasonable and necessary. In other words, the line search technique generates a positive constant $\alpha_{k}$ in a finite frequency of backtracking repetitions. By the established conclusion we propose the following theorem on the global convergence of the proposed algorithm. □

### Theorem 6.1

*If Assumption* 2 *holds and the relevant sequences* $\{d_{k}, \alpha_{k}, x_{k+1},h_{k+1}\}$ *are calculated using Algorithm *5.1, *then*

6.6 $$ \liminf_{k \rightarrow \infty } \Vert h_{k}\Vert =0. $$

### Proof

We prove this by contradiction. This means that there exist a constant $\varepsilon_{0} > 0$ and an index $k_{0}$ such that

$$\Vert h_{k}\Vert \geq \varepsilon_{0}, \quad \forall k \geq k_{0}. $$

On the one hand, by (6.2) and (6.4) we obtain

6.7 $$ \Vert d_{k}\Vert \leq \bigl(\eta_{1}+2(1- \eta_{1})/\eta_{2}\bigr)\Vert h_{k}\Vert \leq \bigl(\eta _{1}+2(1-\eta_{1})/\eta_{2}\bigr) \theta,\quad \forall k \in \Psi. $$

On the other hand, from (6.3) we have

6.8 $$ \Vert d_{k}\Vert \geq \eta_{1}\Vert h_{k} \Vert \geq \eta_{1}\theta. $$

These inequalities indicate that the sequence of $\{d_{k}\}$ is bounded. This means that there exist an accumulation point $d^{*}$ and the corresponding infinite set $N_{1}$ such that

$$\lim_{k \rightarrow \infty }d_{k} =d^{*},\quad k \in N_{1}. $$

By Lemma 6.1 we obtain that the sequence of $\{x_{k}\}$ is bounded. Thus, there exist an infinite index set $N_{2} \subset N_{1}$ and an accumulation point $x^{*}$ that meet the formula

$$\lim_{k \rightarrow \infty } x_{k}=x^{*}, \quad \forall k \in N _{2}. $$

By Lemmas 6.1 and 6.2 we obtain

$$\alpha_{k}\Vert d_{k}\Vert \rightarrow 0,\quad k \rightarrow \infty. $$

Since $\{d_{k}\}$ is bounded, we obtain

6.9 $$ \lim_{k \rightarrow \infty }\alpha_{k}=0. $$

By the definition of $\alpha_{k}$ we obtain the following inequality:

6.10 $$ -h\bigl(x_{k}+\alpha_{k}^{*}d_{k} \bigr)^{T}d_{k} \leq \sigma \alpha_{k}^{*} \bigl\Vert h\bigl(x_{k}+\alpha_{k}^{*}d_{k} \bigr)\bigr\Vert \Vert d_{k}\Vert ^{2}, $$

where $\alpha_{k}^{*}=\alpha_{k}/\rho $. Now, we take the limit on both sides of (6.10) and (6.3) and obtain

$$h\bigl(x^{*}\bigr)^{T}d^{*}>0 $$

and

$$h\bigl(x^{*}\bigr)^{T}d^{*} \leq 0. $$

The obtained contradiction completes the proof. □

## The results of nonlinear equations

In this section, we list the relevant numerical results of nonlinear equations and present the objective function $h(x)=(f_{1}(x), f_{2}(x), \ldots, f_{n}(x))$, where the relevant functions’ information is listed in Table 1.

### Problems and test experiments

To measure the efficiency of the proposed algorithm, in this section, we compare this method with (1.10) (as Algorithm 6) using three characteristics “NI”, “NG”, and “CPU” and the remind that Algorithm 6 is identical to Algorithm 5.1. “NI” presents the number of iterations, “NG” is the calculation frequency of the function, and “CPU” is the time of the process in addressing the tested problems. In Table 1, “No” and “problem” express the indices and the names of the test problems.

*Stopping rule:* If $\|g_{k}\| \leq \varepsilon $ or the whole iteration number is greater than 2000, the algorithm stops.

*Initiation:* $\varepsilon =1e{-}5$, $\sigma =0.8$, $s=1$, $\rho =0.9$, $\eta_{1}=0.85$, $\eta_{2}=\eta_{3}=0.001$, $\eta_{4}= \eta_{5}=0.1$.

*Dimension:* 3000, 6000, 9000.

*Calculation environment:* The calculation environment is a computer with 2 GB of memory, a Pentium(R) Dual-Core CPU E5800@3.20 GHz, and the 64-bit Windows 7 operation system.

The numerical results with the corresponding problem index are listed in Table 4. Then, by the technique in [49], the plots of the corresponding figures are presented for two discussed algorithms.

**Table 4:** Numerical results

NO	Dim	Algorithm 5.1			Algorithm 6		
		NI	NFG	CPU	NI	NFG	CPU
1	3000	161	162	3.931225	146	147	4.149627
1	6000	126	127	12.760882	115	116	11.122871
1	9000	111	112	22.464144	99	100	19.515725
2	3000	5	76	1.185608	5	76	1.060807
2	6000	6	91	4.758031	5	76	4.009226
2	9000	5	62	6.926444	5	62	6.754843
3	3000	33	228	3.276021	18	106	1.778411
3	6000	40	275	15.490899	18	106	6.084039
3	9000	40	285	33.243813	18	106	12.54248
4	3000	4	61	0.842405	4	61	0.936006
4	6000	4	47	2.698817	4	61	3.322821
4	9000	4	47	5.226033	4	61	6.817244
5	3000	23	237	3.244821	23	237	3.354022
5	6000	25	263	14.133691	25	263	13.930889
5	9000	26	278	30.186193	26	278	30.092593
6	3000	1999	29986	382.951255	1999	29986	365.369942
6	6000	88	1307	68.141237	1999	29986	1484.240314
6	9000	65	962	101.806253	1999	29986	3113.998361
7	3000	4	47	0.748805	3	46	0.624004
7	6000	4	47	2.589617	3	46	2.386815
7	9000	4	47	5.257234	3	46	5.054432
8	3000	25	156	2.854818	17	142	1.872012
8	6000	32	189	10.826469	18	162	8.377254
8	9000	28	192	21.512538	19	174	18.938521
9	3000	10	151	1.934412	5	76	1.014007
9	6000	4	61	3.510023	5	76	3.884425
9	9000	4	61	6.614442	6	91	9.609662
10	3000	1999	29986	386.804479	1999	29986	359.816306
10	6000	1999	29986	1523.068963	1999	29986	1469.59182
10	9000	1999	29986	3164.339884	1999	29986	3087.712193
11	3000	498	7457	98.32743	499	7472	93.101397
11	6000	498	7457	385.026068	499	7472	367.787958
11	9000	498	7457	794.07629	498	7457	774.825767
12	3000	1999	2000	51.059127	1999	2000	46.238696
12	6000	1999	2000	199.322478	1999	2000	185.71919
12	9000	1999	2000	405.680601	1999	2000	391.234908
13	3000	1	2	0.0312	1	2	0.0624
13	6000	1	2	0.156001	1	2	0.187201
13	9000	1	2	0.140401	1	2	0.249602
14	3000	1999	29972	400.220565	1999	29973	362.671125
14	6000	1999	29972	1544.316299	1999	29973	1460.294161
14	9000	1999	29972	3197.287295	1999	29973	3105.168705
15	3000	4	61	0.733205	4	61	0.733205
15	6000	4	61	3.790824	4	61	3.026419
15	9000	4	61	6.552042	4	61	6.146439
16	3000	5	62	1.060807	5	62	0.858006
16	6000	5	62	3.400822	5	62	3.291621
16	9000	5	62	6.942044	5	62	6.25564
17	3000	6	77	1.326009	6	91	1.216808
17	6000	6	77	4.243227	6	91	4.570829
17	9000	6	77	8.548855	6	91	9.40686
18	3000	5	76	0.936006	5	76	0.920406
18	6000	5	76	3.900025	5	76	3.775224
18	9000	5	76	8.533255	5	76	7.86245
19	3000	108	1060	15.5689	141	1272	17.565713
19	6000	81	788	44.429085	114	1029	53.820345
19	9000	63	628	70.512452	100	903	99.715839

### Results and discussion

**Figure 4:**
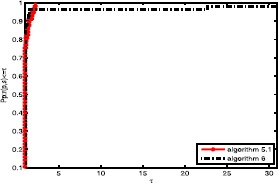
Performance profiles of these methods (NI)

**Figure 5:**
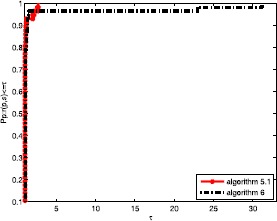
Performance profiles of these methods (NG)

**Figure 6:**
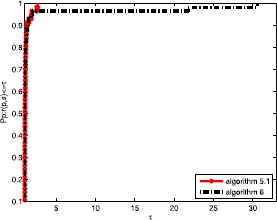
Performance profiles of these methods (CPU time)

From the above figures, we safely arrive at the conclusion that the proposed algorithm is perfect compared to similar optimization methods since the algorithm (1.10) is perfect to a large extent. In Fig. 4 we see that the proposed algorithm quickly arrives at a value of 1.0, whereas the left one slowly approaches 1.0. This means that the objective method is successful and efficient for addressing complex problems in our life and work. It is well known that the calculation time is one of the most essential characteristics in an evaluation index of the efficiency of an algorithm. From Figs. 5 and 6, it is obvious that the two algorithms are good since their corresponding point values arrive at 1.0. This result expresses that the above two algorithms solve all of the tested problems and that the proposed algorithm is efficient.

## Conclusion

This paper focuses on the three-term conjugate gradient algorithms and use them to solve the optimization problems and the nonlinear equations. The given method has some good properties.

(i) The proposed three-term conjugate gradient formula possesses the sufficient descent property and the trust region feature without any conditions. The sufficient descent property can make the objective function value be descent, and then the iteration sequence $\{x_{k}\}$ converges to the global limit point. Moreover, the trust region is good for the proof of the presented algorithm to be easily turned out.
(ii) The given algorithm can be used for not only the normal unstrained optimization problems but also for the nonlinear equations. Both algorithms for these two problems have the global convergence under general conditions.
(iii) Large-scale problems are done by the given problems, which shows that the new algorithms are very effective.
